# Predictors of acute hospital mortality from a 23-year database of emergency medical admissions

**DOI:** 10.1007/s11845-025-04022-2

**Published:** 2025-07-29

**Authors:** Richard Conway, Declan Byrne, Deirdre O’Riordan, Bernard Silke

**Affiliations:** Department of Internal Medicine, St James’s Hospital, Dublin 8, Ireland

**Keywords:** 30-day mortality, Acute medicine, Long-term survival

## Abstract

**Background:**

There is much focus on the management of the acute emergency medical admission; however, comparative outcome data over long time periods are sparse.

**Aim:**

We report all the outcomes of emergency admissions over 23 years (2002–2024).

**Methods:**

Demographic and clinical details are described. Predictors of 30-day in-hospital mortality were analysed with logistic and Cox regression.

**Results:**

The 2002–2024 cohort consisted of 186,004 admissions in 95,192 patients. The 30-day in-hospital mortality per admission averaged 3.8% (95% CI 3.7% to 3.9%); there was a relative risk reduction (RRR) between 2002 and 2024 of 55.3%, from 5.5% to 2.4% (*p* = 0.001), with a calculated number needed to treat (NNT) of 33. Per patient mortality (single admission only considered; last admission if > 1) averaged 7.5% (95% CI 7.4% to 7.7%); there was a RRR of 77.9% between 2002 and 2024, from 13.1% to 2.9% (*p* = 0.001) with NNT of 9.9. Total in-hospital deaths were 14,092 at respective age for males of 73.6 (SEM 0.12) and females of 79.4 (SEM 0.11). The strongest predictors of short-term 30-day in-hospital mortality were being older (> 70 years)—OR 2.05 (95%CI 1.94, 2.18) and the acute illness severity score 1.91 (95%CI 1.85, 1.96). Other predictors were the Charlson Index 1.32 (95% CI 1.28, 1.37) and a primary neurological MDC (major disease category) 1.18 (95% CI 1.09, 1.27).

**Conclusion:**

The 30-day in-hospital mortality for emergency medical admissions has improved over time.

## Introduction

The introduction of acute medical units (AMU) improved outcomes, with a significant reduction in 30-day in-hospital mortality after their implementation [1]. In recent years, hospitals have seen a steady rise in emergency admissions, driven by older persons with multiple comorbidities, on polypharmacy or having atypical clinical presentations leading to 75% remaining too long in the emergency department (ED) [2]. The AMU structured intake facilitates quicker assessments, diagnoses, and treatments that have reduced the length of hospital admissions and eased pressures on ED [1, 3, 4]. The general applicability of the model has been debated [2]. The impact of the level of experience in managing the admission is uncertain; practitioners with greater cumulative clinical experience/skill might enhance outcomes—however, the evidence is conflicting [5].

We have previous published acute hospital outcomes up to 15 years [6–8], with a linear improvement in mortality outcomes over time. Important predictive outcomes of the acute hospital mortality were the acute illness severity score (AISS), derived from admission laboratory data [9], and the pre-existing co-morbidity burden, as quantified by the comorbidity score [10]. In the current study, we were interested to see if previously reported improvements in outcomes over time would continue. We now analyse the outcomes over more than two decades utilising a database of all emergency medical admissions to St. James’ University Hospital, Dublin, over a 23-year period (2002–2024).

## Methods

### Background

St James’s Hospital, Dublin, serves as a secondary care centre for emergency admissions in a catchment area with a population of 270,000 adults. All emergency medical admissions are admitted from ED to an AMU, the operation and outcome of which have been described elsewhere [11, 12].

### Data collection

An anonymous patient database was employed, assembling core information from each clinical admission including details from the patient administration system, national hospital in-patient enquiry (HIPE) scheme, the patient electronic record, and laboratory data. HIPE is a national database of coded discharge summaries from acute public hospitals in Ireland [13]. The International Classification of Diseases, Ninth Revision, Clinical Modification (ICD-9-CM) has been used for both diagnosis and procedure coding from 1990 to 2005 and ICD-10-CM since then. Data included parameters such as the unique hospital number, admitting consultant, date of birth, gender, area of residence, principal and up to nine additional secondary diagnoses, principal and up to nine additional secondary procedures, and admission and discharge dates. Additional information cross-linked and automatically uploaded to the database includes physiological, haematological, and biochemical parameters. This study includes all emergency medical admissions between 2002 and 2024. The study received institutional ethics approval.

### Risk predictors

Derangement of admission biochemical parameters may be utilised to predict clinical outcome. We have previously derived and applied an AISS [9], predicting 30-day in-hospital mortality from parameters recorded in the ED [14]. A weighted age adjusted score was derived; six risk groups (I–VI) were identified with initial cut-points for 30-day in hospital mortality set at 1, 2, 4, 8, and 16%.

### Comorbidity score

Patient morbidity was assessed by a comorbidity score [10] published in 2014, which was further adjusted by additional information collected by our information system since [15]. To devise the score, we searched ICD codes that captured chronic physical or mental health disorders that limit people in activities that they generally would be expected to be able to perform were grouped according to the following ten systems: (i) cardiovascular, (ii) respiratory, (iii) neurological, (iv) gastrointestinal, (v) diabetes, (vi) renal, (vii) neoplastic disease, (viii) others (including rheumatological disabilities), (ix) ventilatory assistance required, and (x) transfusion requirement. In addition, we searched our hospital’s other databases for evidence of diabetes (Diamond database) [16], respiratory insufficiency (FEV1 < 2L), troponin status (high sensitivity troponin ≥ 25 ng/L) [17], low albumin (< 35 G/dL), and anaemia (haemoglobin levels < 10 G/dL) or chronic renal insufficiency—MDRD < 60 mL/min*1.73 m2 [18]. Each component of the score was then weighted according to 30-day in-hospital mortality.

### Statistical methods

Descriptive statistics were calculated for background demographic data, including means/standard deviations (SD), medians/inter-quartile ranges (IQR), or percentages. Comparisons between categorical variables and mortality were made using chi-square tests. We adjusted the outcome computation (30-day in-hospital mortality) for other known predictor variables including AISS [9, 20], comorbidity score [15, 21], and blood culture status [22]. We employed a logistic model with robust estimate to allow for clustering; the correlation matrix thereby reflected the average discrete risk attributable to each of these predictor variables [9].

Logistic regression analysis identified potential mortality predictors and then tested those that proved to be significant univariate predictors (*p* < 0.1 by Wald test) to ensure that the model included all variables with predictive power. We used the margins command in Stata to estimate and interpret adjusted predictions for sub-groups, while controlling for other variables such as time, using computations of average marginal effects. Margins are statistics calculated from predictions of a previously fitted model at fixed values of some covariates and averaging or otherwise over the remaining covariates. In the multivariable logistic regression model, we adjusted univariate estimates of effect, using the previously described outcome predictor variables. Survival regression calculations were undertaken employing Cox’s proportional hazards model, for two groups with the assumption of constant between-group hazard function over time. Testing for the equality of survivor functions was with the log-rank test.

Adjusted odds ratios (OR) and 95% confidence intervals (CI) were calculated for those predictors that significantly entered the model (*p* < 0.10). Statistical significance at P < 0.05 was assumed throughout. Stata v.17.0 (Stata Corporation, College Station, Texas) statistical software was used for analysis.

## Results

### Patient demographics

There were a total of 186,004 emergency medical admissions in 95,192 unique patients in the 23 years between January 2002 and October 2024. This included patients admitted directly into the intensive care unit or high dependency unit. The proportion of males was 48.2%. The median (IQR) LOS was 4.1 (1.2, 9.3) days. The median (IQR) age was 60.5 (43.3, 74.8) years, with the upper 10% boundary at 84.6 years.

### Demographics related to 30-day in-hospital mortality (Table 1)

**Table 1:** Emergency medical admissions (2002–2024)—admission characteristics

Variable		Reference	30-day death	*p*-value
*N*		186,004	14,092	
Age (yr.)		60.5 (43.3, 74.8)	78.5 (67.9, 85.3)	< 0.001
LOS (days)		4.1 (1.2, 9.3)	9.3 (3.8, 24.6)	< 0.001
Gender	Male	80,214 (52.1%)	6938 (49.4%)	< 0.001
	Female	73,890 (47.9%)	7116 (50.6%)	
Acute Illness Severity Score	1—3	71,714 (46.5%)	1769 (12.6%)	< 0.001
	4	22,672 (14.7%)	1195 (8.5%)	
	5	23,141 (15.0%)	2262 (16.1%)	
	6	36,577 (23.7%)	8866 (62.9%)	
Charlson Index	0	83,491 (54.2%)	4550 (32.4%)	< 0.001
	1	40,286 (26.1%)	3569 (25.4%)	
	2	30,327 (19.7%)	5935 (42.2%)	
Comorbidity Score	< 6	117,435 (76.2%)	7775 (55.2%)	< 0.001
	6 < 10	34,795 (22.6%)	5641 (40.0%)	
	> = 10	1874 (1.2%)	676 (4.8%)	

There was a highly significant difference between the ages of the initial survivors 60.5 (43.3, 74.8) and 30-day in-hospital deaths 78.5 (67.9, 85.3) (*p* < 0.001). There were similar differences in the shorter hospital LOS for survivors 4.1 (1.2, 9.3) compared with the initial hospital death cohort 9.3 (3.8, 24.6) (*p* < 0.001). Those who died had higher AISS, comorbidity score, and Charlson Index at admission.

**Fig. 1:**
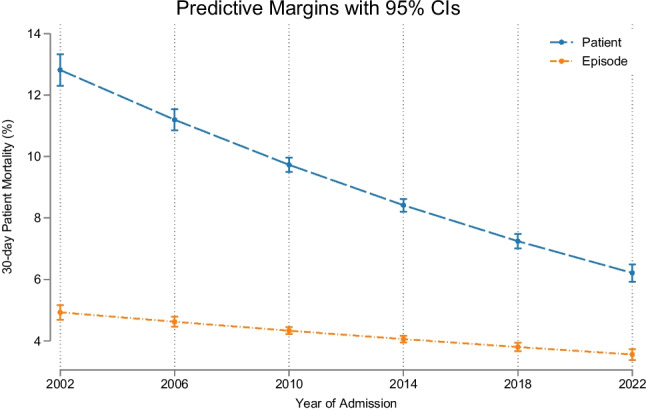
30-day in-hospital mortality calculated per admission episode and per patient from the multivariable logistic regression model. The predicted probabilities were derived from and plotted based on the model prediction. There was a consistent trend for modelled outcomes (adjusted for major outcome predictors) to improve over time

Over time, the 30-day in-hospital mortality improved significantly, with per patient mortality (single admission only considered; last admission if > 1) over the 23 years averaging 7.5% (95%CI 7.4% to 7.7%); there was a relative risk reduction (RRR) of 77.9% between 2002 and 2024, from 13.1% to 2.9% (*p* = 0.001) (Fig. 1), with number needed to treat (NNT) of 9.9. Calculated per admission 30-day in-hospital mortality over the 23 year period averaged 3.8% (95% CI 3.7% to 3.9%); there was a RRR of 55.3% between 2002 and 2024, from 5.5% to 2.4% (*p* = 0.001), with a NNT of 33.

**Fig. 2:**
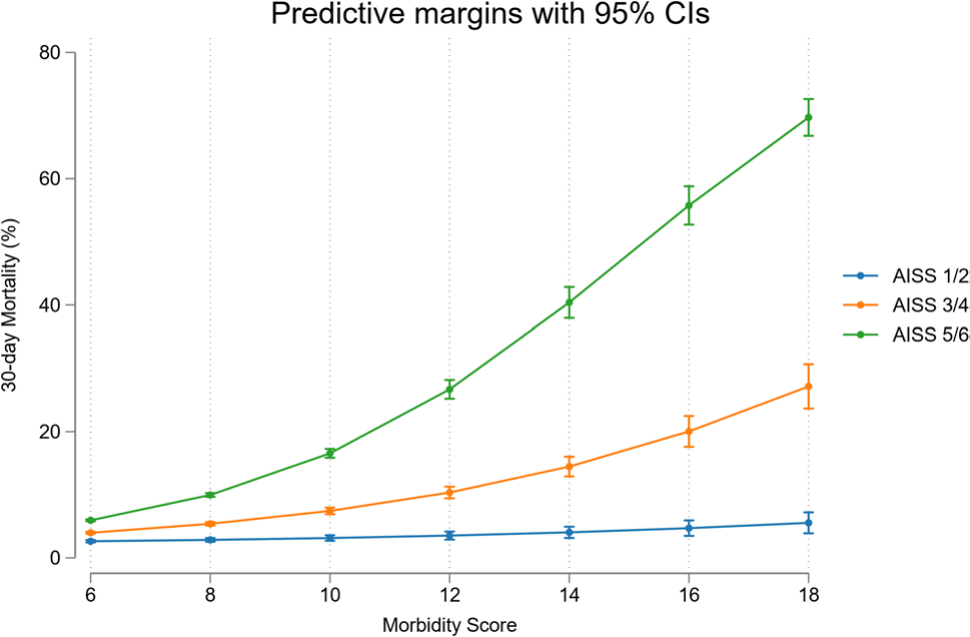
30-day in-hospital mortality from the multivariable logistic regression model with illustration of the AISS and comorbidity score interaction. At higher comorbidity scores there was increasing relationship between these predictor variables

The strongest predictors of short-term 30-day in-hospital mortality were being older (> 70-years)—OR 2.05 (95% CI 1.94, 2.18) and the AISS 1.91 (95% CI 1.85, 1.96). Other less strong predictors were the Charlson Index 1.32 (95% CI 1.28, 1.37) and a primary neurological MDC (major disease category) 1.18 (95% CI 1.09, 1.27). There was an interaction between the AISS and comorbidity burden; the mortality risk increases initially as non-linear function (~ exponential increase related to number of comorbidities) but then approaches a linear function at high comorbidity scores (Fig. 2).

The total number of in-hospital deaths was 14,092 with average age for males of 73.6 (SEM 0.12) and females of 79.4 (SEM 0.11); this represents a shortfall of survival, from the projected references of 82.5 (male) and 85.3 (female), with respective life-year loss of 8.9 years and 5.9 years. There are a total of 74 electoral divisions in the hospital catchment area, with deprivation ranks of quintile I (*n* = 13), quintile III (*n* = 5), quintile IV (*n* = 4), and quintile V (*n* = 49), based on SAHRU classification [19]. Clearly the large majority (~ 70%) of catchment admissions, with this demographic pattern, will be from a low SES (socio-economic status) population.

## Discussion

This paper describes the improvement in outcome of the acute emergency medical admission over the past two decades. The AMU model must at least in part take some of the credit; such developments occurred in tandem with the strengthening of governance systems and quality improvement processes, with the AMU having become an important site for improvement in the care of patients with acute illness [4]. The association between consultant experience on patient outcome following AMU admission remains uncertain and deserves further study; however, supporting and retaining experienced clinicians in the front-line in acute patient care is vital—if hospital system pressures are to be addressed [5]. This point is vital as we consider the implications of a shift to 7-day-operations, and the qualitative and quantitative staffing impacts. The philosophy of the AMU may often lack clarity, and there may be conflict between organisational management seeking efficiency, the physician’s goal of promoting healing, and the emerging professional dogma to ‘get things right first time’ [23]. Often, considering the uncertainty in medicine and the range of possible variations in clinical presentations, expectations on outcomes in modern society, no matter what the circumstance, are set at a level that is unachievable. Nonetheless, these data provide considerable grounds for optimism; with the caveat that endless progress delivered by quality improvement must have finite limits.

Our catchment consists of ~ 70% deprived areas, whose life expectancy can be anticipated to be considerably shortened. However, over time, the first observation was the remarkable improved outcomes over two decades. Considering the in-hospital 30-day mortality outcome over 23 years and counting only the last admission if more than one had occurred, there was a RRR of 77.9% between 2002 and 2023, from 13.1% to 2.9% (*p* = 0.001), with NNT of 9.9. Even just considering all admissions, the RRR was still 55.3% from 5.5% to 2.4% (*p* = 0.001), with a NNT of 33. This remarkable improvement occurred despite disrupted continuity, with an increasing number of disparate clinicians without prior knowledge of the case contributing to the acute call roster, and it can possibly be partially attributed to improved systems, patient profiling, and accurate imaging.

As with any study design, there are certain limitations inherent in our current work. Our study was conducted in a single large academic teaching hospital; the external validity of our results requires validation in other centres and settings. While our database is comprehensive, containing large amounts of clinically relevant data, it is possible, although we judge unlikely, that residual confounders remain which we have not adjusted.

In conclusion, we demonstrate significant improvements in 30-day in-hospital mortality over two decades. Outcomes are influenced by patient and disease-specific factors. The strongest predictors of 30-day in-hospital mortality were age, illness severity, and comorbidity.

## Data Availability

No further data is available for sharing. All pertinent data has been published in this manuscript.
